# New echinocandin susceptibility patterns for nosocomial *Candida albicans* in Bogotá, Colombia, in ten tertiary care centres: an observational study

**DOI:** 10.1186/s12879-015-0840-0

**Published:** 2015-02-28

**Authors:** Giovanni Rodríguez-Leguizamón, Alessandro Fiori, Katrien Lagrou, María Antonia Gaona, Milciades Ibáñez, Manuel Alfonso Patarroyo, Patrick Van Dijck, Arley Gómez-López

**Affiliations:** School of Medicine and Health Sciences, Universidad del Rosario, Bogotá, Colombia; Faculty of Natural and Mathematical Sciences, Universidad del Rosario, Bogotá, Colombia; VIB Department of Molecular Microbiology, KU Leuven, Leuven, Belgium; KU Leuven Laboratory of Molecular Cell Biology, Leuven, Belgium; Department of Microbiology and Immunology, KU Leuven, Leuven, Belgium; Molecular Biology and Immunology Department, Fundación Instituto de Inmunología de Colombia (FIDIC), Carrera 50#26-20, Bogotá, Colombia

**Keywords:** *Candida albicans*, Nosocomial infection, Susceptibility, Echinocandins, Resistance

## Abstract

**Background:**

*Candida albicans* remains as the first cause of nosocomial fungal infections in hospitals worldwide and its susceptibility pattern should be better described in our tertiary care hospitals.

**Methods:**

This study aimed at identifying the caspofungin susceptibility pattern regarding nosocomial *Candida albicans* infection in ten tertiary care hospitals using the methodology proposed by CLSI M27-A3 and CLSI M27-S4, and its association with risk factors and clinical outcome. The approach involved descriptive research concerning the diagnosis of nosocomial infection during a 7-month period in 10 hospitals in Bogotá, Colombia. Associations were established using exact non-parametric statistical tests having a high statistical power (>95%), suitable for small samples. The exact Mann Whitney test or Kruskall-Wallis non-parametric ANOVA tests were used for distributions which were different to normal or ordinal variables when comparing three or more groups. Multivariate analysis involved using binomial, multinomial and ordinal exact logistical regression models (hierarchical) and discrimination power was evaluated using area under the ROC curve.

**Results:**

101 nosocomial infections were found in 82,967 discharges, for a *Candida* spp. infection rate of 12.2 per 10,000 discharges, 30.7% caused by *C. albicans*, 22.8% by *C. tropicalis*, 20.8% by *C. parapsilosis*, 19.8% by other *Candida*, 3% by *C. krusei* and 3% by *C. glabrata*. Statistically significant associations between mortality rate and the absence of parenteral nutrition were found in multivariate analysis (OR = 39.746: 1.794-880.593 95% CI: p = 0.020). The model’s predictive power was 83.9%, having an 85.9% significant prediction area (69.5%-100 95% CI; p = 0.001).

**Conclusions:**

Significant differences were found regarding susceptibility results when comparing CLSI M27-A3 to CLSI M27-S4 when shifting clinical break-point values. However, one nosocomial strain was consistent in having reduced susceptibility when using both guidelines without having been directly exposed to echinocandins beforehand and no mutations were found in the *FKS1* gene for hot spot 1 and/or hot spot 2 regions, thereby highlighting selective pressure regarding widespread antifungal use in tertiary healthcare centres. Nutritional conditions and low family income were seen to have a negative effect on survival rates.

## Background

*Candida* spp*.* is the leading opportunistic mycotic agent worldwide and it is the fourth cause of bloodstream infections since the 1990s [1,2]. If the rate of mortality due to nosocomial candidaemia were to be specifically noted, it would have given a 49% attributable mortality rate (38%-60% 95% CI), this being very high for an infectious disease, even surpassing the patients’ base disease mortality rate [3].

Continuous advances in medical techniques have also imposed fresh challenges on the management and control of nosocomial fungal infections [4]. According to reports collected over a six-and-a-half-year period of epidemiological surveillance at global level by the ARTEMIS study, *C. albicans* resistance to azoles was distributed as follows: 1.3% fluconazole resistance and 1.0% to voriconazole (however, the figures for Colombia are 6.1% for fluconazole and 4.0% for voriconazole) [5]. Regarding the echinocandins, global studies have been found which have not considered the level of resistance to be significant, given that echinocandins have only recently been introduced into clinical practice [6].

The variability in minimum inhibitory concentration (MIC) (break-points reported in different studies) [6-8], as well as clinical reports of therapeutic failure in patients having MIC results of susceptible strains [9,10] have led to suggesting that break-points for defining susceptibility against echinocandins should be modified [11]. These antecedents motivated our group’s interest in determining nosocomial *C. albicans* strains’ susceptibility pattern in tertiary care hospitals in Bogotá, Colombia.

## Methods

### Study design

This observational study was carried out in ten tertiary care hospitals (each having between 200 and 500 hospital beds); three of these hospitals admit paediatric population only, whilst the remaining ones admit the general population. The nosocomial infection rate was 3.38% in non–ICU wards and 8.26% in ICUs; samples were collected during seven months, giving a total of 77,763 regular hospital discharges in non-ICU ward and 5,204 ICU discharges. The ICUs were mixed purpose (i.e. caring for surgical, coronary, septic and poly-traumatised patients). All cases of nosocomial *Candida* spp*.* infection were reported by infection committees and according to the CDC definition [12]. Ethics committees from the following institutions approved this study: Clínica del Niño, Clínica de Occidente, Fundación Cardioinfantil, Hospital de Kennedy, Fundación Homi-Hospital de la Misericordia, Hospital del Tunal, Hospital Simón Bolívar, Hospital de la Samaritana, Clínica Infantil Colsubsidio, Clínica Colsubsidio Orquídeas and Universidad del Rosario.

All the clinical records were reviewed by each hospital’s nosocomial infection committee regarding patients reported as being cases of nosocomial *Candida* spp*.* infection during 7 months and for whom prior culture results were available. Evident infection 48 hours after having been admitted to hospital supported by a positive microbiological culture was defined as a case of nosocomial infection, registered in the clinical record by the treating physician. The treating physician defined whether the clinical diagnosis gave either invasive candidiasis or urinary tract infection by *Candida* spp*.* based on clinical interpretation, laboratory reports and positive cultures [12]. Clinical records were verified by medical researchers for all patients and the information was recorded in an Excel database and Epi Info (version 6.0). All clinical samples were collected as part of standard patient care and later managed by a centralised laboratory for verifying *Candida* spp*.* presence.

### Antifungal susceptibility test

A broth micro-dilution test (BMD) was carried out at KU Leuven’s Molecular Cell Biology laboratory according to the indications given in the CLSI M27-A3 document [13]. The new recommendations made in CLSI M27-S4 guidelines were incorporated in additional assays; such assays were carried out in the Corporación de Investigaciones Biológicas (CIB) microbiology laboratory in Medellín, following all guideline quality and reproducibility recommendations [14].

### Quality control

Quality was controlled according to CLSI indications using recommended *C. krusei* ATCC 6258 and *C. parapsilosis* ATCC 22019 strains [13].

### Antifungal drugs

Caspofungin (Merck), micafungin (Astellas) and fluconazole (Sigma) were used in this study. The working solution for each antifungal drug was prepared in sterile water following CLSI M27–A3 guidelines, the first two dilutions in water and the final dilutions in RPMI 1640 solution adjusted to pH 7.0 with 0.165 M morpholinepropanesulfonic acid (MOPS) buffer [13]. All *C. albicans* nosocomial isolates were tested with micafungin dissolved in DMSO, following CLSI M27-S4 recommendations.

### Microorganisms

One hundred and one nosocomial *Candida* spp. strains were isolated throughout the study, 31 of them being nosocomial *C. albicans*. These isolates were obtained from the epidemiological surveillance being carried out by each hospital infection committee and corresponded to nosocomial infection criteria; each infection recorded corresponded to an episode per patient.

### Identifying the microorganisms

Colombian clinical isolates were initially typed using manual microbiological systems (API32C and germ tube) and those classified as being *C. albicans* were further confirmed by using MALDI Biotyper RTC software 3.0 (Bruker); the identification spectrum was obtained in duplicate and compared to the MSP reference spectrum, simultaneously selected from the BDAL software database (Bruker) and the CBS–KNAW library. The classification results obtained by MALDI-TOF-MS (spectrum identification *cf* MSP) led to assigning a score in line with the equipment’s requirements and classified as follows: firm identification of genus and specie (≥2.0), reliable identification of genus (1.7–2.0) and non-reproducible identification (≤1.7) [15].

### Analysing *FKS1* gene sequencing

*FKS1* gene sequences were amplified using previously described primers for hot spot (HS) 1 (HS1) and 2 (HS2) regions: F 5′ GTTCCACCAGTTTATACATTCC 3′ and 5′ ATGTCACTCTTGAGAATTGATC 3′ R for HS1 and F 5′ GCTCATGAAGCTATCATGTGTT 3′ and 5′ CAAGACAAACACCTAAACATCC 3′ R for HS2 [10,16]. All reactions involved using KAPA HiFi containing 0.3 μM of each primer in a final 25 μL volume. Thermal conditions were set as follows: denaturing at 95°C for 5 min, followed by 35 cycles of denaturing at 98°C for 30 seconds, primer annealing at 54°C for 20 seconds and an extension step at 72°C for 1 min followed by a final extension step at 72°C for 5 min. Attempts were made to characterise mutations on at least two different occasions, forming part of two subsequent biological repetitions; the amplification products were purified by using a Promega Wizard purification systems kit and then sent for sequencing in both directions (5′ and 3′) with a BigDye Terminator kit (Macrogen, Seoul, South Korea).

### Statistical analysis

The data was processed using SPSS (version 20.0) and STATA (version 11.0) software.

Absolute and relative frequency distribution was expressed as percentages for qualitative variables and measurements of central tendency, the average and mean dispersion, the range and standard deviation were used for quantitative variables. The association of susceptibility and mortality with different factors, Fisher’s exact test and likelihood ratio were evaluated for qualitative variables and Shapiro Wilk’s test was used for evaluating normality in normal variables, Student’s *T*-test for average differences for independent groups having homogenous or heterogeneous variability, using Levene’s test for evaluating the homogeneity of variance. The exact Mann Whitney test or Kruskall-Wallis non-parametric ANOVA tests were used for distributions which were different to normal or ordinal variables when comparing three or more groups. Multivariate analysis involved using binomial, multinomial and ordinal exact logistical regression models (hierarchical) and discrimination power was evaluated using area under the ROC curve. Statistical tests were evaluated using 5% and 10% significance.

## Results

### Patients’ characteristics

One hundred and one nosocomial *Candida* spp. infections were identified during 7 months from 77,763 non-ICU ward discharges and 5,204 ICU discharges, giving an incidence rate of 12.2 nosocomial fungaemia per 10,000 discharges; distribution was 30.7% *C. albicans*, 22.8% *C. tropicalis*, 20.8% *C. parapsilosis*, 19.8% other *Candida spp.*, 3% *C. krusei* and 3% *C. glabrata*. The distribution of nosocomial *C. albicans* infection showed that 7 clinical isolates (13%) came from non-ICU ward discharges and 24 clinical isolates (87%) from ICUs; Table 1 shows the related clinical characteristics. No significant differences were found regarding gender distribution (51.6% females and 48.4% males; p = 1.00, exact binomial test). In age groups known as being at risk of acquiring infection, 8 patients (26%) aged less than 1 year old and 7 patients (22%) older than sixty were found. There was 33% mortality, similar to that described in the literature [3]. Diagnosis for nosocomial infection caused by *Candida* spp*.* had a similar distribution between invasive candidiasis (58.1%) and urinary tract infection (41.9%) caused by *C. albicans*. Regarding risk factors, more than four broad-spectrum antibiotics were being used at the same time (10 patients: 32.3%), central venous catheter use (25 patients: 80.6%), parenteral nutrition (18 patients: 58.1%) and more than 15 days’ hospital length of stay (27 patients: 87%).

**Table 1:** **Demographic and clinical characteristics**

**Variable**	**ICU n cases (%)**	**Non-ICU ward n cases (%)**
**Age ≤ 1 year**	7 (23%)	1 (3%)
**Age ≥ 60 years**	5 (16%)	2 (6%)
**Male gender**	13 (42%)	2 (6%)
**Invasive Candidiasis**	14 (45%)	4 (13%)
**UTI**	10 (32%)	3 (10%)
**Mortality**	7 (23%)	3 (10%)
**Broad spectrum AB use**	24 (77%)	6 (19%)
**CVC**	21 (68%)	4 (13%)
**Abdominal surgery**	6 (19%)	4 (13%)
**TPN**	15 (48%)	3 (10%)
**Immunosuppressant**	6 (19%)	0
**Length of stay ≥15 days**	22 (71%)	5 (16%)

Note: ICU: intensive care unit, UTI: urinary tract infection, AB: antibiotic, CVC: central venous catheter, TPN: total parenteral nutrition.

### Antifungal susceptibility

Table 2 shows MIC values for determining caspofungin susceptibility following the standardised antifungal susceptibility test, according to CLSI M27-A3 indications for the *C. albicans* group previously identified by MALDI-TOF-MS. Table 2 also shows the MIC values used for determining micafungin susceptibility, according to the new CLSI M27-S4 recommendations. The GV608 strain was resistant to micafungin following the CLSI M27-S4 guidelines and had previously shown resistance to caspofungin following CLSI M27-A3 guidelines.

**Table 2:** **Antifungal susceptibility to caspofungin according to CLSI M27-A3 and micafungin according to CLSI M27-S4**

**Strain**	**MIC CSF**	**MIC MCF**
**SC5314**	≤0.25 μg/mL (S)	≤0.25 μg/mL (S)
**GV475**	≥1 μg/mL (R)	≤0.25 μg/mL (S)
**GV78**	0.5 μg/mL (I)	≤0.25 μg/mL (S)
**GV527**	2 μg/mL (R)	≤0.25 μg/mL (S)
**GV242**	≥1 μg/mL (R)	≤0.25 μg/mL (S)
**GV147**	≥1 μg/mL (R)	≤0.25 μg/mL (S)
**GV608**	**≥1 μg/mL (R)**	**0.5 μg/mL (R)**

Note: MIC: minimal inhibitory concentration, CSF: caspofungin, MCF: micafungin, S: susceptible, I: intermediate, R: resistant.

The isolate resistant to both caspofungin and micafungin is shown in bold.

### Characterising the *FKS1* gene mutation pattern

The sequencing results were analysed using CLUSTALW software and compared to GenBank reference sequences (accession No. D88815). No mutations were found in the GV608 nosocomial strain for the aforementioned regions after analysing both resistant strain sequences.

### Statistical association

There was a significant association between mortality and low socioeconomic level, mortality being greater in patients having a daily income of less than 9€ (50.0%, 9/18 *cf* 15.4%, 2/13) (p = 0.05, Fisher’s exact test). *C. albicans* infected patients with no parenteral nutrition had a significant association with mortality when compared to those having parenteral nutrition (61.8%, 8/13 *cf* 16.7%, 3/18) (p = 0.014, Fisher’s exact test).

The multivariate exact unconditional logistical regression model for mortality was significantly associated with the absence of parenteral nutrition (OR = 39.746: 1.794- 880.593 95% CI; p = 0.020) and closely associated with a background of being undernourished (p = 0.079, OR = 14.21). The other factors were not significant (having an abdominal surgery during hospital stay, the number of antibiotics or length of hospital stay). The model’s discrimination power was 83.9% (85.9% area under the ROC curve: 69.5-100 95% CI; p = 0.001).

No statistically significant associations were found between the susceptibility pattern and the clinical outcomes.

## Discussion

The present study explored the *C. albicans* infection pattern regarding nosocomial infection and led to characterising susceptibility patterns in a Colombian hospital setting. This was the first study of its kind to be carried out in a tertiary care hospital network in Bogotá; it also explored the echinocandin susceptibility profile as a recent therapeutic alternative for managing patients at risk of infection by this fungus. There were differences between CLSI M27-A3 and CLSI M27-S4 guidelines as the clinical break-points shifted. CLSI M27-S4 gave less strains having reduced susceptibility; however, a nosocomial strain was consistently micafungin-resistant, without having been directly exposed to echinocandins.

The other relevant findings concerned the clinical associations found regarding this type of nosocomial infection, as well as aspects related to the conditions of patients requiring nutritional support and having a low monthly income background, thereby affecting the clinical outcome.

Our study focused on providing a description of *Candida* spp. nosocomial infection; a high incidence rate of 12.2 cases of infection per 10,000 discharges was revealed in a representative sample of 10 tertiary care hospitals, accounting for 82,967 total discharges over a 7-month period. This finding contrasted with that in the pertinent literature which referred to nosocomial fungaemia incidence of 4.9 cases of infection per 10,000 discharges [17], thereby revealing greater nosocomial infection incidence caused by this microorganism in the sample of hospitals in Bogotá. Taking the total amount of nosocomial infections caused by *Candida* spp.*,* distribution by species maintained the frequency described in previous studies in Colombia, where *Candida albicans* occupied first place regarding frequency (30.7%) [18]; however, this is the first report made in Colombia which has evaluated clinical and microbiological aspects referring to a representative population involved in nosocomial infection from the 10 tertiary care hospitals which participated in the study.

The echinocandins are currently first-line treatment agents when managing patients having had previous exposure to azoles, a report of *Candida* spp*.* involving reduced susceptibility to azoles and/or patients in a critical or unstable clinical condition [19]. Caspofungin is the drug of choice for patients having the aforementioned characteristics; its use is widespread due to it being the first molecule approved by the FDA [20,21].

According to epidemiological surveillance studies carried out around the world, excellent levels of activity for echinocandins *in vitro* have been observed during a six year follow-up, suggesting that emergent resistance to echinocandins is hardly likely since the caspofungin inhibition percentage for *C. albicans* was 99.6% [22]. However, later studies have shown the need for re-evaluating the break-points defining echinocandin susceptibility as clinical observations have been made regarding therapeutic failure, in spite of susceptibility tests having indicated lower than 2 μg/mL values. Such break-points were adjusted for defining susceptibility as ≤0.25 μg/mL, intermediate susceptibility as 0.5 μg/mL and ≥1 μg/mL for resistance [11]. A recent review of clinical break-points proposed that just ≤0.25 μg/mL should be used for susceptibility and ≥0.5 μg/mL for resistance [23]. Micafungin or anidulafungin use has recently been proposed instead of caspofungin in susceptibility tests because of variability regarding the break-points obtained by several laboratories [24]. Caspofungin is the most used antifungal agent in clinical practice and the correlation between *in vitro* break-points for micafungin regarding patients being treated with caspofungin thus merits new studies. Our study revealed differences between both CLSI guidelines, indicating that the M27-S4 guideline recommendations should be followed in new studies. It is worth stressing that just one nosocomial strain was consistently micafungin-resistant regarding both methodologies and had not been previously exposed to echinocandins. It should be noted that mutations in the *FKS1* gene for HS1 and/or HS2, as described in the pertinent literature, were not found for this nosocomial strain [10]. The foregoing shows the need for adjusting clinical break-points, as the susceptibility pattern regarding *Candida* spp*.* infections is dynamic and is influenced by patient and strain exposure to prolonged treatment with different antifungal drugs currently available on the market [9,10,25]. Even though the molecular definition of resistance to echinocandins has led to identifying punctual mutations in the *FKS1* gene [16,20,26], other authors have managed to identify low susceptibility to echinocandins in the absence of such mutations [27,28]. The foregoing involves other mechanisms, meaning that other alternatives could be considered for explaining the reduced susceptibility pattern observed in our isolate [27,28]. This *C. albicans* strain could have represented a nosocomial infection transmission pattern where, according to prior reports, healthcare workers hands represent the main vehicle of transmission [29].

It is worth noting that this is the first observational study reported in a population having a higher nosocomial fungal infection rate (12.2/10,000 discharges) than that reported in pertinent literature (4.9/10,000 discharges [17]) which highlights the importance of the results here found, despite the relatively low sample size. However, associations were established using exact non-parametric statistical tests having a high statistical power (>95%), suitable for small sample sizes [30,31] to overcome this problem.

This is the first study which has evaluated a representative nosocomial sample of *C. albicans* infection and the first aimed at adapting and parametrising the CLSI M27-S4 guidelines for Colombia. Neither case had a report of having received prior antifungal treatment and this also had not been described previously in the literature; these results reinforce the concept of nosocomial fungal transmission and the microbiological outcomes highlight the need of carrying out combination drug therapy studies in order to minimise resistance risk [32,33]. Additional microbiological and molecular studies are required for describing this susceptibility pattern; this would lead to a better understanding of the mechanisms facilitating these microorganisms becoming adapted to exposure to such antifungal drugs.

Another relevant aspect concerning studying *C. albicans* infection concerns the clinical factors associated with a risk of contracting the infection [34]. In spite of some reports having indicated an increase in infections caused by non-*albicans* species, *C. albicans* continues being responsible for most cases involving fungal infections in hospitals [35], besides, the associated risk factors have not changed. The overall description of patients suffering *C. albicans* infection in our study has shown that the presence of central venous catheter, prior broad spectrum antibiotic use and parenteral nutrition had the greatest association [36], as well as most patients having stayed in an ICU.

Regarding the statistical analysis of 31 patients having nosocomial *C. albicans* infection, significant associations were identified which were related to the two most relevant variables: mortality and nutritional condition.

This study revealed an association between mortality and patients having a daily income of less than 9€, this being difficult to find in the pertinent literature and represents an external factor concerning the quality of hospital care which could affect an outcome which is unfortunately associated with a particular social condition. The Colombian healthcare system offers equal coverage to all population. Associated factors such as the type of institution or haemodynamic state which could have affected or been associated with this variable were also explored but their values were not significant (i.e. the level of medical treatment was equitable for all patients).

Regarding bivariate analysis of patients infected by *C. albicans*, another strongly associated factor referred to the absence of parenteral nutrition and mortality when contrasting our study with the literature related to mortality studies. This factor was not specifically mentioned [3], though references were made to the presence of parenteral nutrition and a risk of candidaemia [37]. However, some important work refers to nutritional state and infection and how compromise regarding nutritional state favours the risk of infection and even a patient becoming immunocompromised [38]. Multivariate analysis covering all the risk factors for mortality and candidaemia described in different work [3,37] was taken into account when designing an exact unconditional logistical regression multivariate model which led to finding a significant association between the absence of parenteral nutrition. This indicated the relevance of including patients’ nutritional state and the relevance of including suitable nutritional support for providing their needs in a clinical evaluation.

Several studies have been published about nosocomial *Candida* spp*.* infection presentation mode and frequency, as well as regarding the risk factors associated with the infection [3,37]; however, little information is available regarding patients’ susceptibility, socioeconomic conditions and nutritional support. The nosocomial infection rate found in the present study was representative of the samples taken in the tertiary care hospitals which participated in the study and was even higher than that reported in the literature [17]. The relevance of these results has emphasised aspects regarding prevention and the rational use of antifungal therapeutic schemes, expressed by other authors as being one of the best strategies for controlling this emergent problem [39,40].

## Conclusions

Some differences were found regarding susceptibility results comparing CLSI M27-A3 to CLSI M27-S4 when shifting clinical break-point values. However, one nosocomial strain was consistent in having reduced susceptibility when using both guidelines without having been directly exposed to echinocandins beforehand and had no mutations in regions previously described for the *FKS1* gene, thereby indicating selective pressure concerning widespread antifungal use in tertiary healthcare centres. Nutritional conditions and low family income were seen to have a negative effect on survival rates. The clinical findings documented regarding the first echinocandin-resistant nosocomial strain in Colombia which has not been exposed to this antifungal drug indicate the urgent need for epidemiological surveillance studies regarding susceptibility patterns following the latest CLSI M27-S4 recommendations.

## Abbreviations

CLSI: Clinical Laboratory Standards Institute
ROC Curve: Receiver Operating Characteristic Curve
MIC: Minimum Inhibitory Concentration
ICU: Intensive Care Unit
CDC: Centers for Disease Control and Prevention
BMD: Broth Micro-Dilution test
MOPS: Morpholinepropanesulfonic acid buffer
MALDI: Matrix-Assisted Laser Desorption/Ionization
MSP: Main Spectrum
MALDI-TOF-MS: Matrix-Assisted Laser Desorption/Ionization Time Of Flight Mass Spectrometry
FDA: Federal Drug Administration
UTI: Urinary Tract Infection
AB: Antibiotic
CVC: Central Venous Catheter
TPN: Total Parenteral Nutrition
